# Herpes zoster in patients with sciatica

**DOI:** 10.1186/s12891-020-03847-5

**Published:** 2020-12-05

**Authors:** Der-Shin Ke, Chao-Yu Hsu, Cheng-Li Lin, Chung-Y. Hsu, Chia-Hung Kao

**Affiliations:** 1 Department of Medical Education, Ditmanson Medical Foundation Chia-Yi Christian Hospital, Chia-Yi, Taiwan; 2 Department of Optometry, Central Taiwan University of Science and Technology, Taichung, Taiwan; 3 Center for General Education, National Taichung University of Science and Technology, Taichung, Taiwan; 4 Department of General Education, National Chin-Yi University of Technology, Taichung, Taiwan; 5 The General Education Center, Chaoyang University of Technology, Taichung, Taiwan; 6 Center for General Education, National Chi Nan University, Puli, Taiwan; 7 Rural Generalist Program Japan, GENEPRO, Chiba, Japan; 8 Management Office for Health Data, China Medical University Hospital, Taichung, Taiwan; 9 College of Medicine, China Medical University, Taichung, Taiwan; 10 Graduate Institute of Biomedical Sciences and School of Medicine, College of Medicine, China Medical University, No. 2, Yuh-Der Road, Taichung, 404 Taiwan; 11 Department of Nuclear Medicine and PET Center, China Medical University Hospital, Taichung, Taiwan; 12 Department of Bioinformatics and Medical Engineering, Asia University, Taichung, Taiwan; 13 Center of Augmented Intelligence in Healthcare, China Medical University Hospital, Taichung, Taiwan

**Keywords:** Sciatica, Herpes zoster, Depression, Obesity

## Abstract

**Background:**

Several diseases are associated with herpes zoster (HZ). However, whether sciatica is a stressor leading to HZ development remains unclear. Here, we evaluated the occurrence of HZ in patients with sciatica.

**Methods:**

The sciatica cohort consisted of patients first diagnosed as having sciatica between 2000 and 2012. All patients with sciatica were randomly age, sex and index year matched with control individuals without sciatica. The primary outcome was diagnosis of HZ. All individuals were followed until HZ diagnosis, withdrawal from the insurance, death, or December 31, 2013, whichever occurred first. HZ risk in the two cohorts was further analyzed with age, sex and comorbidity stratification.

**Results:**

In total, 49,023 patients with sciatica and 49,023 matched controls were included. Female patients were more likely to have HZ development than were male patients [adjusted hazard ratio (HR) = 1.07, 95% confidence interval (CI) = 1.02–1.12]. After adjustments for all the covariates, HZ risk was significantly higher in the sciatica cohort than in the control cohort (adjusted HR = 1.19; 95% CI = 1.12–1.25).

**Conclusion:**

Sciatica increased HZ risk. Thus, HZ risk should be addressed whenever physicians encounter patients with sciatica, HZ vaccination should be considered especially those aged over 50.

## Background

Sciatica is characterized by a burning sensation or shooting pain caused by irritation or compression of the sciatic nerve, and the most common cause of sciatica is spinal disc herniation. Sciatica prevalence and incidence rates have been reported to be 1.2–43% and 1–37%, respectively; the large variations may be due to the differences in definitions or study population in the concerned studies [1, 2]. Sciatica treatment intensity is dependent on disease severity, and the treatment can include exercise, manual therapy, medication, spinal injection and surgery [3].

Herpes zoster (HZ), a painful vesicular eruption, occurs due to varicella-zoster virus (VZV) reactivation. HZ incidence was 9.92 per 1000 person-years in immunocompetent, unvaccinated adults aged ≥50 years, with a case-fatality rate of 0.04% [4]. HZ incidence can be high after transplantation. Kim et al. reported that HZ incidence after liver transplantation in adults was 16.3 per 1000 person-years. HZ incidence was 9.1, 10.0, and 11.9% at 3, 5, and 10 years after transplantation, respectively [5]. Postherpetic neuralgia is a painful complication; Salvetti et al. found that one-tenth of HZ patients suffered from this, with the highest proportion of 15.56% being found in patients aged 75–79 years [6].

Diseases with chronic pain such as adhesive capsulitis of the shoulder [7], chronic interstitial cystitis [8], and lateral epicondylitis [9] are associated with HZ occurrence. Sciatica is one of the diseases with chronic pain, the association between sciatica and HZ development might be existed. In this paper, we evaluated HZ occurrence in patients with sciatica to understand whether sciatica is a stressor leading to HZ development.

## Methods

### Study design and research database

We designed a population-based retrospective cohort study. Here, the study data were extracted from the claims data in the Longitudinal Health Insurance Database 2000 (LHID2000); which includes systemically collected claims data of 1 million National Health Insurance (NHI) as well as the random samples in Taiwan NHI Research Database (NHIRD). NHIRD, managed by the National Health Research Institutes, contains data of beneficiaries of the NHI program established in 1995 to provide comprehensive and universal health care coverage to approximately 99% Taiwan residents. NHIRD include data on enrollment files, claims data, disease diagnose, prescriptions, outpatient visits, and hospital admissions. All diagnoses are coded using International Classification of Diseases, Ninth Revision, Clinical Modification (ICD-9-CM) diagnostic codes. To ensure data privacy, patient data are released to researchers in an electronically encrypted form and thus, the requirement to obtain informed consent was waived. This study has been approved by the Research Ethics Committee at China Medical University Hospital (CMUH104-REC2–115-CR-4).

### Data availability statement

The dataset used in this study is held by the Taiwan Ministry of Health and Welfare (MOHW). The MOHW must approve our application to access this data. Any researcher interested in accessing this dataset can submit an application form to the Ministry of Health and Welfare requesting access. Please contact the staff of MOHW (Email: stcarolwu@mohw.gov.tw) for further assistance. Taiwan Ministry of Health and Welfare Address: No.488, Sec. 6, Zhongxiao E. Rd., Nangang Dist., Taipei City 115, Taiwan (R.O.C.). Phone: + 886–2–8590-6848. All relevant data are within the paper.

### Study population

We identified patients first diagnosed as having sciatica (ICD-9-CM 724.3) between January 1, 2000 and December 31, 2012, and included them in the sciatica cohort. The index date was defined as the date of sciatica diagnosis. We excluded patients aged < 20 years, and with a HZ history before the index date. Every patient with sciatica was randomly age (every 5-year interval), sex and index date matched with a control individual without sciatica from the same database and then this individual was assigned to the control cohort under the same criteria.

### Outcome and covariate assessment

The outcome of interest was a new diagnosis of HZ (ICD-9-CM 053) between January 1, 2000 and December 31, 2013. All individuals were followed until HZ occurrence, withdrawal from NHI, death, or December 31, 2013, whichever occurred first. We considered several covariates as potential confounders including sex, age, and baseline comorbidities. Inpatient and outpatient data were used to define the status of comorbidities including chronic kidney disease (ICD-9-CM 585and 586), obesity (ICD-9-CM 278), diabetes (ICD-9-CM 250), coronary artery disease (ICD-9-CM 410–414), depression (ICD-9-CM 296.2, 296.3, 300.4 and 311), and lumbar disc herniation (ICD-9-CM 722.10).

### Statistical analysis

Characteristics of all included individuals were first analyzed descriptively. The differences of categorical and continuous variables between the sciatica and control cohorts were tested using the *t* and chi-square tests. The incidence rate was defined as the number of events per 1000 person-years. Cox proportional hazards regression model adjusted for age, sex and comorbidity was used to determine the association between sciatica and HZ risk. The results were presented as a hazard ratio (HR) with accompanying 95% confidence interval (CI). We estimated the cumulative incidence of HZ in the sciatica and control cohorts by using the Kaplan–Meier method, and the differences were examined using the log-rank test. HZ risk in the two cohorts was further analyzed after age, sex and comorbidity stratification. A two-sided *p* of < 0.05 was considered statistically significant. All data processing and statistical analyses were performed using SAS (version 9.4; SAS Institute Inc., Cary, NC, USA).

## Results

In total, 49,023 patients with sciatica and 49,023 matched controls were included. Table 1 presents the demographic characteristics and comorbidities in the two cohorts. The age and sex distribution was similar between the sciatica and control cohorts after matching. Compared with the control cohort, the sciatica cohort had significantly higher diabetes, coronary artery disease, depression, obesity, cancer and lumbar disc herniation prevalence (*p* < 0.001). The mean follow-up duration was 7.44 (±3.82) and 7.42 (±3.83) years in sciatica and control cohorts, respectively.

**Table 1:** Demographic characteristics and comorbidities in cohorts with and without Sciatica

Variable	Sciatica		*p*-value
	No	Yes	
	*N* = 49,023	*N =* 49,023	
Age, year			0.99
≤ 49	18,729 (38.2)	18,729 (38.2)	
50–64	16,682 (34.0)	16,682 (34.0)	
65+	13,612 (27.8)	13,612 (27.8)	
Mean ± SD^a^	54.1 ± 15.4	54.8 ± 15.1	< 0.001
Sex			0.99
Female	27,378 (55.9)	27,378 (55.9)	
Male	21,645 (44.2)	21,645 (45.2)	
Comorbidity			
Diabetes	3849 (7.85)	4592 (9.37)	< 0.001
CAD	7491 (15.3)	11,260 (23.0)	< 0.001
Depression	2016 (4.11)	3356 (6.85)	< 0.001
Chronic kidney disease	859 (1.75)	926 (1.89)	0.11
Obesity	544 (1.11)	932 (1.90)	< 0.001
Cancer	1376 (2.81)	1178 (2.40)	< 0.001
Lumbar disc herniation	16,769 (34.2)	38,692 (78.9)	< 0.001

Chi-Square Test; ^a^: T-Test

*CAD* denotes coronary artery disease

Cox proportional hazards regression models for analyzing the risk of variables contributing to HZ are presented in Table 2. After adjustment for all the covariates, the sciatica cohort had a significantly higher HZ risk than did the control cohort (adjusted HR = 1.19; 95% CI = 1.12–1.25). Moreover, HZ risk was significantly higher in patients aged 50–64 years (adjusted HR = 2.10; 95% CI = 1.97–2.23) and those aged > 65 years (adjusted HR = 2.48; 95% CI = 2.32–2.66) than in those aged < 49 years. Female patients were more likely to have HZ than were male patients (adjusted HR = 1.07, 95% CI = 1.02–1.12). Moreover, patients with diabetes, coronary artery disease, depression, chronic kidney disease, cancer and lumbar disc herniation had a significantly higher HZ risk than did those without any comorbidity.

**Table 2:** The incidence and risk factors for herpes zoster

Variable	Event	PY	Rate^a^	Crude HR(95% CI)	Adjusted HR^b^ (95% CI)
Sciatica					
No	2899	368,778	7.86	1.00	1.00
Yes	3981	375,977	10.6	1.35 (1.28, 1.41)***	1.19 (1.12, 1.25)***
Age, year					
≤ 49	1551	310,651	4.99	1.00	1.00
50–64	2839	254,722	11.2	2.25 (2.11, 2.39)***	2.10 (1.97, 2.23)***
65+	2490	179,382	13.9	2.84 (2.67, 3.03)***	2.48 (2.32, 2.66)***
Sex					
Female	4192	426,137	9.84	1.16 (1.11, 1.22)***	1.07 (1.02, 1.12)*
Male	2688	318,618	8.44	1.00	1.00
Comorbidity					
Diabetes					
No	6113	690,290	8.86	1.00	1.00
Yes	767	54,465	14.1	1.61 (1.50, 1.74)***	1.14 (1.05, 1.12)**
CAD					
No	5039	615,072	8.19	1.00	1.00
Yes	1841	129,683	14.2	1.75 (1.66, 1.85)***	1.18 (1.12, 1.25)***
Depression					
No	6455	709,775	9.09	1.00	1.00
Yes	425	34,980	12.2	1.36 (1.23, 1.50)***	1.11 (1.01, 1.23)*
Chronic kidney disease					
No	6727	735,561	9.15	1.00	1.00
Yes	153	9193	16.6	1.88 (1.60, 2.20)***	1.28 (1.09, 1.50)**
Obesity					
No	6788	735,209	9.23	1.00	1.00
Yes	92	9546	9.64	1.06 (0.87, 1.31)	
Cancer					
No	6691	730,816	9.16	1.00	1.00
Yes	189	13,939	13.6	1.51 (1.31, 1.75)***	1.21 (1.05, 1.40)*
Lumbar disc herniation					
No	2538	348,796	7.28	1.00	1.00
Yes	4342	395,959	11.0	1.53 (1.46, 1.61)***	1.24 (1.17, 1.31)***

Rate^a^, incidence rate, per 1000 person-years; Crude HR, relative hazard ratio; Adjusted HR^b^: multivariable analysis including age, sex, and comorbidities of diabetes, and CAD;

*p < 0.05, **p < 0.01, ***p < 0.001

**Fig. 1:**
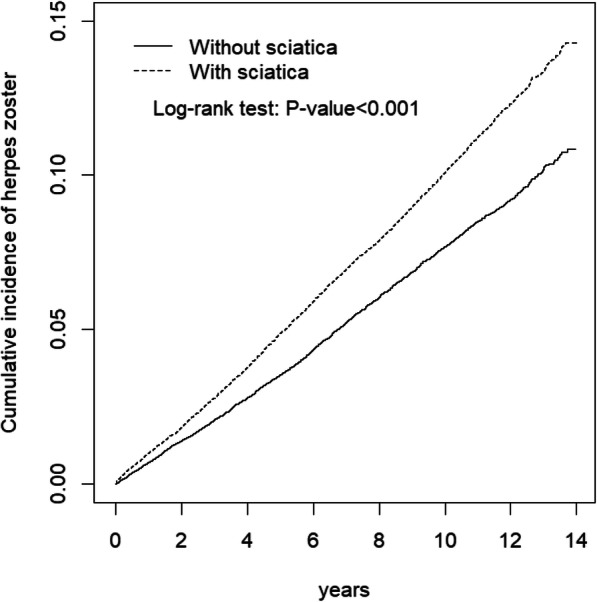
Cummulative incidence comparison of herpes zoster for patients with (dashed line) or without (solid line) sciatica

Table 3 presents the stratification analysis results. After age, sex and comorbidity stratification, HZ incidence was significantly higher in the sciatica cohort than in the control cohort (*p* < 0.001). Similarly, the Kaplan-Meier analysis results revealed that the cumulative incidence of HZ was significantly different between the sciatica and control cohorts (log-rank test, *p <* 0.001; Fig. 1).

**Table 3:** Incidence of herpes zoster by age, sex and comorbidity and Cox model measured hazards ratio for patients with Sciatica compared those without Sciatica

Variables	Sciatica						Crude HR (95% CI)	Adjusted HR^b^ (95% CI)
	No			Yes				
	Event	PY	Rate^a^	Event	PY	Rate^a^		
Age, years								
≤ 49	662	154,980	4.27	889	155,671	5.71	1.34 (1.21, 1.48)***	1.17 (1.04, 1.31)***
50–64	1174	127,099	9.24	1665	127,623	13.1	1.41 (1.31, 1.52)***	1.30 (1.19, 1.41)***
65+	1063	86,700	12.3	1427	92,682	15.4	1.25 (1.16, 1.36)***	1.11 (1.02, 1.21)***
Sex								
Female	1758	211,353	8.32	2434	214,784	11.3	1.36 (1.28, 1.45)***	1.21 (1.14, 1.30)***
Male	1141	157,426	7.25	1547	161,192	9.60	1.32 (1.23, 1.43)***	1.14 (1.04, 1.24)***
Comorbidity ^c^								
No	1242	213,614	5.81	586	73,197	8.01	1.35 (1.22, 1.49)***	1.37 (1.24, 1.51)***
Yes	1657	155,164	10.7	3395	302,780	11.2	1.04 (0.98, 1.11)	1.14 (1.08, 1.21)***

Rate^a^, incidence rate, per 1000 person-years; Crude HR, relative hazard ratio; Adjusted HR^b^: multivariable analysis including age, sex, and comorbidities of diabetes, CAD, and lumbar disc herniation;

^c^Individuals with any comorbidity of diabetes, CAD, depression, and chronic kidney disease, obesity, cancer, and lumbar disc herniation were classified into the comorbidity group

***p < 0.001

Similar results were observed for HZ by propensity score methods as sensitivity analysis. We recreated a non-sciatica cohort well matched for age, sex, index year and comorbidities including diabetes, CAD, depression, chronic kidney disease, obesity, cancer, lumbar disc herniation (Table 4). With this newly selected non-sciatica cohort, the associations between sciatica and HZ (adjusted HR = 1.19, 95% CI = 1.12–1.27) remained strongly (Table 5).

**Table 4:** Demographic characteristics and comorbidities in cohorts with and without sciatica by propensity score matching

Variable	Sciatica		*p*-value
	No	Yes	
	*N* = 27,097	*N* = 27,097	
Age, year			0.74
≤ 49	9228 (34.1)	9262 (34.2)	
50–64	9400 (34.7)	9378 (34.6)	
65+	8469 (31.3)	8457 (31.2)	
Mean ± SD^a^	55.8 ± 15.1	55.8 ± 15.2	0.99
Sex			0.99
Female	15,740 (58.1)	15,778 (58.2)	
Male	11,357 (41.9)	11,319 (41.8)	
Comorbidity			
Diabetes	2488 (9.18)	2488 (9.03)	0.55
CAD	5517 (20.4)	5528 (20.4)	0.91
Depression	1438 (5.31)	1457 (5.38)	0.72
Chronic kidney disease	508 (1.87)	458 (1.69)	0.10
Obesity	384 (1.42)	379 (1.40)	0.86
Cancer	772 (2.85)	719 (2.65)	0.16
Lumbar disc herniation	16,769 (61.9)	16,766 (61.9)	0.98

Chi-Square Test; ^a^: T-Test

*CAD* denotes coronary artery disease

**Table 5:** Overall Incidence of herpes zoster (per 1000 person-years) and estimated hazard ratios according to sciatica status using Cox method by propensity score matching

	Sciatica	
	No	Yes
Variable	(*N =* 27,097)	(*N =* 27,097)
Herpes zoster.		
Person-years	203,946	208,742
Follow-up time (y), Mean ± SD	7.53 ± 3.87	7.70 ± 3.84
Event, n	1813	2239
Rate^a^	8.89	10.7
Crude HR (95% CI)	1(Reference)	1.21 (1.13, 1.28)***
Adjusted HR^b^ (95% CI)	1(Reference)	1.19 (1.12, 1.27)***

Rate^a^, incidence rate, per 1000 person-years; Crude HR, relative hazard ratio; Adjusted HR^b^: multivariable analysis including age, sex, and comorbidities of diabetes, and CAD;

***p < 0.001

## Discussion

This is the first population-based study to assess HZ risk in patients with sciatica; patients with sciatica were 1.19 times more likely to develop HZ than were those without.

About 90% of sciatica is caused by spinal disc herniation [10]. Because an inflammatory response in the lumbosacral nerve roots due to herniated nucleus pulposus and mechanical deformation on the nerve, sensation of pain occurs [11]. However, Stafford et al. emphasized that herniation of nucleus pulposus is not the only cause of sciatica, and we should not forget other causes [11].

Diseases with chronic pain such as adhesive capsulitis of the shoulder [7], chronic interstitial cystitis [8], and lateral epicondylitis [9] are associated with risk of HZ. Sciatica is one of common chronic pain syndrome which is a stressor for affected individual. Stress can activate neural or hormonal activity in order to restore homeostasis [12]. It is believed that stress and pain will produce changes in the perceptual and stress system, resulting in abnormal output patterns of the body’s own neuromatrix [12]. These mechanisms are strongly associated with decreasing the VZV-specific cellular immunity; thus, increasing risk of HZ in patients with sciatica should be considered.

In a comprehensive review of the evidence from systematic reviews, Parreira et al. identified risk factors for sciatica from 54 items, with depression being an adverse risk factor [13]. Oosterhuis et al. investigated prognostic factors for work participation in patients with sciatica through systemic reviews and found a similar result: less depression was a favourable factor for return to work [14]. Lower back pain is a discomfort experienced by patients with sciatica. Tutoglu et al. assessed depression severity in patients with sciatica and neuropathic pain, sciatica without neuropathic pain, and healthy participants by using Beck Depression Inventory (BDI) and found mean BDI scores of 5.89 (±5.37), 20.88 (±12.39) and 4.21 (±5.95). BDI was significantly higher in patients with sciatica and neuropathic pain [15]. Max et al. examined depression symptoms by using the 36-items short form of Mental Health Scale for patients with sciatica after surgery and found that depression symptoms were significantly alleviated after surgery if the pain was reduced by > 25%. And, the mean score remained unchanged if there was no or only slight pain relief [16]. The patients with chronic pain may experience depression [17]. Narita et al. reported that chronic pain had an anxiogenic effect in mice and that this phenomenon may be associated with changes in opioidergic function in the amygdala [17]. The mechanisms of pain leading to depression were identified by Max et al. through pain-gene interaction by using a clinical genetic method, they considered that the short-term and 1-year effect on mood after surgery for sciatica was on the mu opioid receptor and galanin-2 receptor [16].

Irwin et al. found that depression is associated with decreasing VZV-specific cellular immunity [18]. Thus, the risk of HZ is increased in patients with depression. Choi et al. found that HZ prevalence was 6.8% in patients with depression and 6.3% in controls and that patients with depression had a 1.09 times higher HZ risk than did those without depression [19]. In a similar population-based study of Liao et al., HZ incidence was 4.58 and 3.54 per 1000 person-years in patients with depression and controls. In addition, their case cohort was 1.11 times more likely to develop HZ than was their control cohort [20]. Because sciatica and sciatica-related conditions such as pain and depression are stressful, there is a strong possibility for sciatica patients to develop HZ.

HZ prevalence typically increases with age as corroborated by our results (Table 1); among our patients with sciatica, HZ incidence was higher in patients aged < 65 years (Table 3). Furthermore, regardless of whether the patients had comorbilities, HZ incidence remained high. These results confirmed that the presence of sciatica might be a stressor leading to HZ development.

### Limitations

This was a retrospective study and thus there were several limitations. First, sciatica severity and HZ severity and location which may affect the treatment decision and prognosis, are unavailable in NHIRD. Second, lifestyles information such as smoking, is also unavailable in the NHIRD. A strongly association between smoking and sciatica was confirmed by several studies [13, 21]. This could also influence the outcome of this study. Third, influence of medication was not analyzed in the present study. Drugs may influence the immune system which resulted to HZ occurrence. However, medication will be given immediately after diagnosis of diseases in both groups due to the unique insurance system with high accessibility, thus the medication bias can be ignored. Fourth, diagnostic bias between different specialists may occur. Nevertheless, all insurance claims are sent to the NHI Administration and reviewed by experts through a strict system of audit and penalty. Therefore, the diagnostic codes are reliable. Despite these limitations, the population-based study provided sufficient evidence for persuasive research through inclusion a large number of patients. Thus, we confirm that HZ risk is higher in patients with sciatica than in those without.

## Conclusion

The presence of sciatica increased HZ risk. Thus, HZ risk should be noted when physicians encounters patients with sciatica, and HZ vaccination should be considered especially those aged over 50.

## Data Availability

The dataset used in this study is held by the Taiwan Ministry of Health and Welfare (MOHW). The Ministry of Health and Welfare must approve our application to access this data. Any researcher interested in accessing this dataset can submit an application form to the Ministry of Health and Welfare requesting access. Please contact the staff of MOHW (Email: stcarolwu@mohw.gov.tw) for further assistance. Taiwan Ministry of Health and Welfare Address: No.488, Sec. 6, Zhongxiao E. Rd., Nangang Dist., Taipei City 115, Taiwan (R.O.C.). Phone: + 886–2–8590-6848. All relevant data are within the paper.

## Abbreviations

HZ: Herpes zoster
HR: Hazard ratio
CI: Confidence interval
VZV: Varicella-zoster virus
LHID2000: Longitudinal Health Insurance Database 2000
NHIRD: National Health Insurance Research Database
ICD-9-CM: International Classification of Diseases, Ninth Revision, Clinical Modification
BDI: Beck Depression Inventory
